# Resilience of Natural Gas Networks during Conflicts, Crises and Disruptions

**DOI:** 10.1371/journal.pone.0090265

**Published:** 2014-03-12

**Authors:** Rui Carvalho, Lubos Buzna, Flavio Bono, Marcelo Masera, David K. Arrowsmith, Dirk Helbing

**Affiliations:** 1 School of Mathematical Sciences, Queen Mary University of London, London, United Kingdom; 2 University of Zilina, Univerzitna 8215/1, Zilina, Slovakia; 3 European Laboratory for Structural Assessment, Institute for the Protection and Security of the Citizen (IPSC), Joint Research Centre, Ispra(VA), Italy; 4 Energy Security Unit, Institute for Energy and Transport, Joint Research Centre, Petten, The Netherlands; 5 ETH Zurich, Zurich, Switzerland; 6 Risk Center, ETH Zurich, Swiss Federal Institute of Technology, Zurich, Switzerland; University of Maribor, Slovenia

## Abstract

Human conflict, geopolitical crises, terrorist attacks, and natural disasters can turn large parts of energy distribution networks offline. Europe's current gas supply network is largely dependent on deliveries from Russia and North Africa, creating vulnerabilities to social and political instabilities. During crises, less delivery may mean greater congestion, as the pipeline network is used in ways it has not been designed for. Given the importance of the security of natural gas supply, we develop a model to handle network congestion on various geographical scales. We offer a resilient response strategy to energy shortages and quantify its effectiveness for a variety of relevant scenarios. In essence, Europe's gas supply can be made robust even to major supply disruptions, if a fair distribution strategy is applied.

## Introduction

Almost everything we do in the course of a day involves the use of energy. Yet, history has taught us that the threats to the security of supply come in unexpected ways [1], [2]. Examples of unforeseen energy crises include the recent disputes between Russia and Ukraine over the price of natural gas (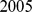
–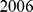, 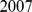
–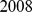, 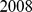
–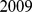) [3], the disruption of the oil and gas production industry in the US following Hurricanes Katrina and Rita (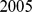) [4], the terrorist attack on the Amenas gas plant that affected more than 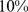 of Algerian production of natural gas (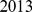) [5], and the supply shortage in March 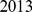, when the UK had only 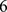 hours worth of gas left in storage as a buffer [6]. New vulnerabilities could come from cyber attacks to the infrastructure [1], particularly in the case of state-driven attacks [2]; be the result of prolonged uncertainty or inaction on energy security in the US or Europe [7]; or derive from an extended period of extremely volatile prices due to intense international conflict [2].

Natural gas, a fossil fuel that accounts for 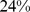 of energy consumption in OECD-Europe [8], has been at the heart of these crises. Gas is expensive to transport, and this is done mainly over a pipeline network. The investments are large and are made with long-term horizons, often of decades, and the costs are covered by locking buyers into long-term contracts [9]. Moreover, current infrastructure investments in Europe still derive from a historical dependency on supply from Russia and North Africa [10]. This dependency leaves the European continent exposed to both a pipeline network that was not designed to transport large quantities of gas imported via Liquefied Natural Gas (LNG) terminals, and to the effects of political and social instabilities in countries that are heavily dependent either on the export of natural gas ( e.g., Algeria, Libya, Qatar or Russia) or its transit ( e.g., Ukraine). Hence, it is challenging to build infrastructure that will be resilient to a wide range of possible crisis scenarios [11].

In a crisis, less delivery may mean greater congestion. This is due to the breakdown of major transit routes or production losses in affected areas, which cause the supply network to be used in different ways from what it was designed for. Hence, the available resources cannot be distributed well with the remaining transport capacities [12] [13], [14]. This is why we need a method to handle congestion.

To manage the gas pipeline network during crises, we propose a decentralized model of congestion control that distributes the available network capacity to each route, without sacrificing network throughput [15]–[17]. A central controller makes the system vulnerable both to attacks on the control centre and to delays and failures of the lines of communication through the network. In contrast, a decentralized method is more resilient to failures because damage to the network has only a local effect and the need for communication is reduced [15], [18]. To illustrate our model, we analyse the throughput of the present and planned pipeline networks across a range of different crisis scenarios at European, country and urban levels. The most challenging scenario corresponds to a hypothetical crisis with Russia with a complete cut-off of supply to Europe. We analyse how to alleviate the impact of such scenarios, by the identification of country groups with similar interests, which should cooperate closely to manage congestion on the network. This acknowledges that many of the 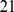
st century challenges, such as the management of energy grids and infrastructure networks [19]–[21], cannot be solved by technology alone, but do have a relevant behavioural or social component [22]–[24].

## Results

### Data set and model

**Figure 1:**
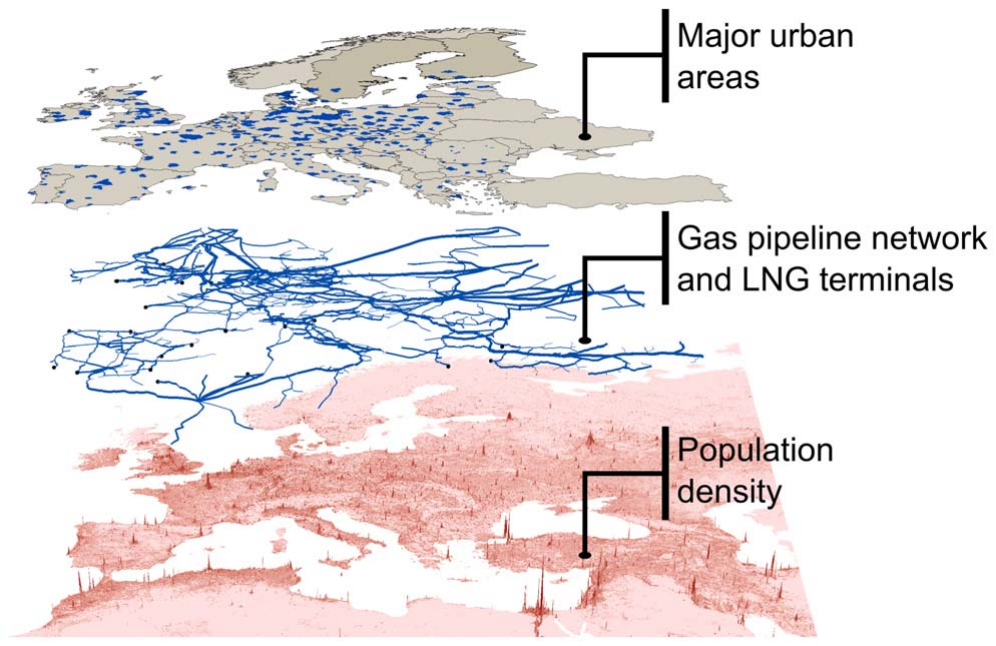
Spatial data layers involved in our analysis: population density (source: Landscan 2012); gas pipeline network and Liquefied Natural Gas (LNG) terminals (source: Platts 2011); and major urban areas (sources: European Environment Agency and Natural Earth). Map composed in ESRI ArcGIS.

**Figure 2:**
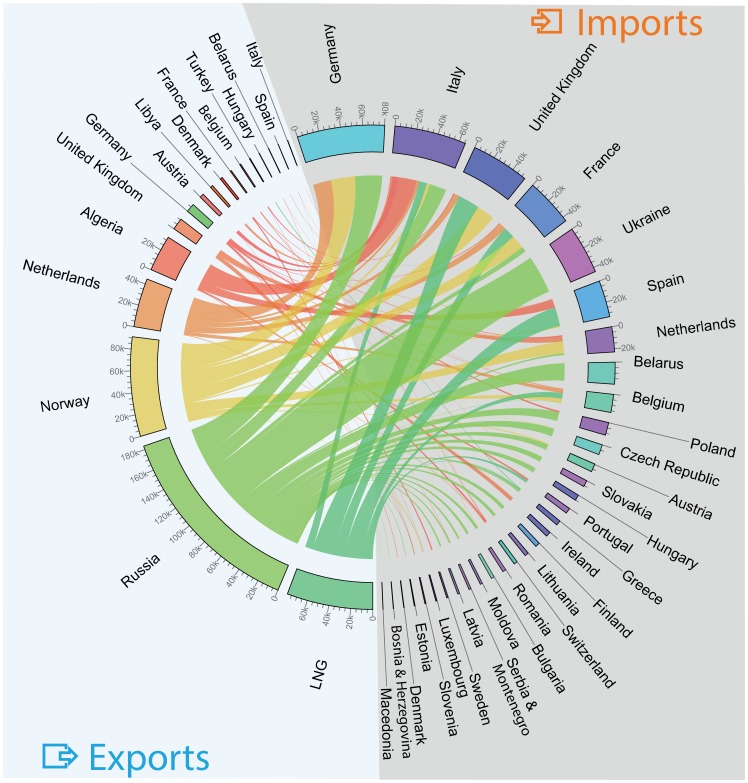
Natural gas imports by pipeline and via Liquefied Natural Gas (LNG) terminals in Europe during 2011 (million cubic meters). Gas exporting (importing) countries are on the left (right) of the image. For each exporting country, we show the breakdown of the volumes of gas exported annually, together with the importing countries served. For each importing country, we show the volumes of gas imported annually, together with the diversity of supply.

Our data set is organized in four layers (see “Databases” in File S1), three of which are shown in Figure 1. The first layer is the population density, which we compute from the 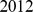 Landscan global population data set. The second layer is the European gas pipeline network and Liquefied Natural Gas (LNG) terminals, which we extract from the Platts 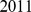 geospatial data set. This infrastructure is a spatial network, where nodes and links are geographically located, and links have capacity and length attributes. The third layer is defined by the urban areas in Europe with 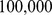 or more inhabitants, and we compile it from the *European Environment Agency* and *Natural Earth*. The fourth layer is the network of annual movements of gas via pipelines and of Liquefied Natural Gas (LNG) via shipping routes (see Figure 2). We represent gas flowing from an exporting country 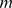 (including LNG) to an importing country 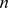, by a directed network with weighted adjacency matrix 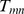.

Gas enters the network at source nodes, is transported over long distances on the pipeline transmission network, and then passed to the distribution network that delivers it to consumers. Here we model only the transport of gas on the transmission network. To model consumption spatially, we first need a tessellation of each country into disjoint sets of urban and non-urban areas, such that the pipeline network in an area is associated with the population it serves. Urban areas are naturally defined by the boundary of their spatial polygons. We partition non-urban areas by a Voronoi tessellation with the gas pipeline nodes as generators, respecting country borders and excluding all urban areas (see “The Model” in File S1).

We assume that the flow of gas on each pipeline intersecting an urban polygon ( i.e., the border of the urban area) is directed towards the centre of the urban area. For simplicity, we also assume that such pipelines supply the urban area from the closest node to the urban polygon that is located inside the urban area. Moreover, each non-urban area is defined by a Voronoi cell, and we assume that it is supplied by the cell generator node (see “The Model” in File S1).

To connect sink to source nodes with paths (see Contract Paths in Methods), we first go through each non-zero entry in the 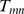 transport matrix and link each sink node in an importing country 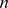 to the 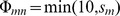 closest nodes in an exporting country 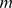, if 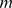 is a country, or to all LNG terminals in country 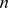, if 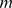 is LNG, where 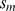 is the number of gas pipeline nodes in an exporting country 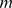.

To allocate demand to individual paths, we start with the assumption that the demand 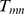 of an importing country 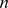 from an exporting country 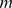 is distributed proportionally to the population of country 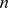 [25]. We next split the demand 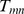 among all source to sink paths between countries 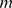 and 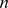, proportionally to the population served by each sink node. We now have a value of demand associated with each path, and therefore with each sink node. Finally, we replace each path by a set of identical paths, each having the minimum demand on the network. This implies that all paths have the same demand, while doubling the demand on a path is equivalent to creating two identical paths with the original demand (see “The Model” in File S1).

To begin integrating routing and congestion control, we first consider how to distribute the capacity 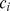 of one single congested link over the 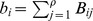 paths that pass through the link, where 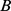 is the link-path incidence matrix (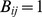 if link 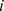 belongs to the path 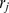 and 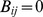 otherwise), and where 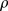 is the number of paths on the network (see Table 1 of the File S1). To find the exact routing for these paths, we apply an iterative algorithm that, for each source-sink pair, finds the path with minimum effective path length, where the effective link length is given by 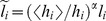, 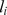 is the length of link 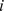, 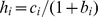, and 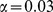 (see “The Model” in File S1).

We consider two baseline scenarios: the present and future networks. The present baseline scenario is the network that has been operational since 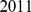; the future baseline scenario extends the present network by the planned and under construction pipelines. To determine the network effects of crises, we analyse a range of scenarios that consist in hypothetically removing exporting ( e.g., Russia) or transit ( e.g., Ukraine) countries from the baseline scenarios. The scenarios are, thus, identified by the baseline (present or future) and the hypothetically removed country. For example, the present Russia scenario is given by the present network excluding Russia, that is removing all entries in the transport matrix 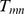 that are movements of gas originating in Russia. Similarly, the future Ukraine scenario is determined by removing all Ukrainian nodes and links from the future network.

Broadly, there are three strategies to manage congestion [26]. First, expanding the network capacity is the most obvious way to lower congestion. The EU has a plan to build major pipelines crossing the continent, that should lower European dependency on Russia (see planned pipelines in Figure S1 of the File S1). Here, we include these planned pipelines in the future scenarios, but make no suggestions for extra infrastructure because the costs of expanding network capacity are high, and thus our focus is on how to best manage the existing and planned network capacity. Second, implementing congestion pricing is a way to cap the consumption of heavy users that cause network bottlenecks. Finally, by identifying groups of importing countries that have similar dependencies on exporting countries, we map a vast number of consumers to a relatively small number of parties that may be able to cooperate during crises [27].

We are aiming at controlling congestion in situations where the network has to perform a function for which it was not designed. For congestion control, we are using the *proportional fairness* algorithm (see Methods), which is inspired by the way capacity is managed on the Internet [15], [28], [29]. This approach could be adjusted to other types of critical infrastructures, such as the power grid and road networks. The main idea behind proportional fairness is to use pricing on the links in order to control congestion (see Methods and “Congestion Control” in File S1). Use of non-congested links is free up to a threshold, above which the cost that a path incurs for using a link increases linearly, but steeply, with the difference between link capacity and link utilization. Hence, paths that traverse many congested links pay a high cost for contributing to congestion, and thus get a smaller flow allocation than paths that avoid congestion. A flow is proportionally fair if, to increase a path flow by a percentage 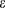, we have to decrease a set of other path flows, such that the sum of the percentage decreases is larger or equal to 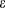. We view the network as an optimizer and the proportional fairness policy as a distributed solution to a global optimization problem [30], [31].

### Simulation Results

For each scenario, we hypothetically remove the scenario country from the network and, if 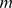 is an exporting country, remove row 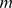 in the 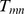 transport matrix. Since the network topology and the flow network 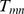 depend on the scenario, we then re-compute the source-sink pairs, the demand of each pair, and we also replace every source-sink path with a number of identical paths, each having the minimum demand in the network. Finally, we apply the proportional fairness congestion control algorithm to the resulting network and paths. We assume that all countries are willing to cooperate, that is, adhere to the rules of the congestion control policy. To assess the effect of the range of scenarios, we then analyse the throughput at the scales of the European continent, countries, and of urban areas.

**Figure 3:**
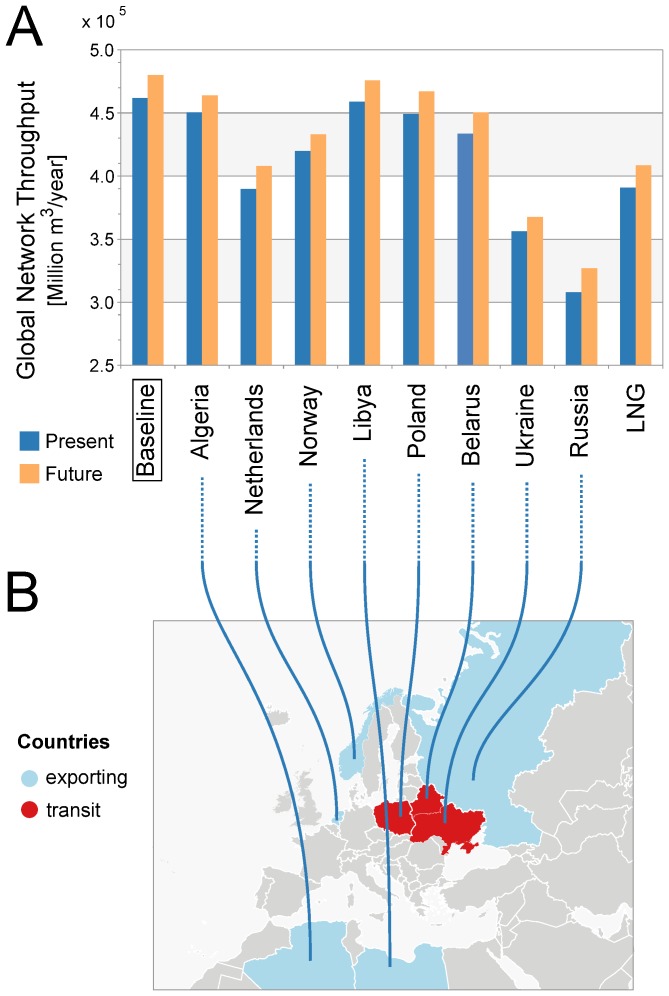
Global network throughput by scenario. (A) A scenario is named after the country that is hypothetically removed from the network, and coloured in blue (orange) if the country is removed from the present (future) baseline scenario. (B) The country removed per scenario is coloured cyan (red) on the map, if it is an exporting (transit) country. The total network throughput increases by 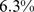 from the present baseline to the future baseline scenario ( i.e., when the future and planned pipelines are added to the present network). The most challenging scenarios are the hypothetical removal of Russia, followed by Ukraine, the Netherlands and LNG. When Russia is removed from the network, the global network throughput falls by 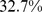 relative to the present baseline and by 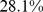 in relation to the future baseline. Figure created from authors' data with ESRI ArcGIS.

We compute the global network throughput, which is the sum of the throughput at all sinks (urban and non-urban), for all the scenarios. Our model reproduces successfully the expected consequences of removing the major source and transit countries from the network: the largest decreases in global network throughput are caused by hypothetically removing Russia, Ukraine, the Netherlands, LNG and Norway (see Figure 3).

**Figure 4:**
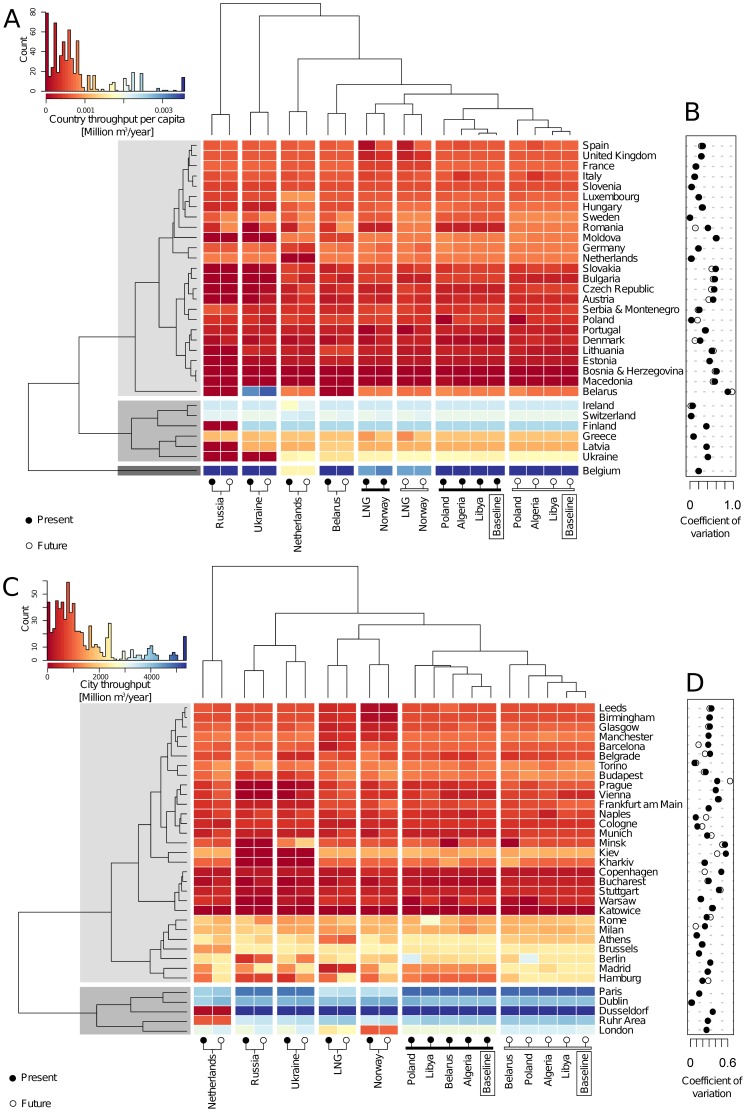
Heat-map [32], illustrating the variation of throughput across various scenarios and the effect of a scenario on the network. The dendrograms are computed using a hierarchical clustering algorithm with the Euclidean norm and average linkage clustering. (A) Heat-map of throughput at country level across various scenarios, allowing for a comparative analysis of the present versus future baseline scenarios, as well as of crises versus baseline scenarios; (B) Coefficient of variation of throughput per capita of a country; (C) Heat-map of throughput at urban level; (D) Coefficient of variation of throughput at urban scale. The gray areas denote groups of countries and urban areas that share common patterns of throughput across scenarios.

We say that a country is resilient to crises if it combines high throughput per capita across scenarios with a low coefficient of variation of throughput. In addition, the network is considered resilient to a scenario if the vectors of country throughput per capita for the scenario and the baseline scenario are similar. To start addressing the resilience of countries and the network to supply and transit crises, we study the signatures in the scenario space given by the country throughput per capita in each of the 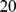 scenarios. Similarly, a scenario can be seen as a point in the 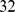-dimensional space of country throughput. The heat-map in Figure 4A shows the throughput per capita for each pair of countries and scenarios [32].

The country groups, determined by dendrograms and highlighted in gray, reflect a similar level of throughput per capita achieved across the scenarios. Countries belong to the high throughput per capita groups (highlighted in dark gray in the figure) due to a combination of effects: diversity of supply; good access to network capacity (strategic geographical location); and a relatively small population (see “Results” in File S1). The coefficient of variation, shown in Figure 4B for present and future scenarios, measures the normalized dispersion of country throughput per capita using the mean as a measure of scale. Larger values indicate that the throughput accessible to a country varies across scenarios. Figure 4B shows that countries in Eastern Europe have high coefficient of variation of throughput per capita in the scenarios where we hypothetically remove Russia or Ukraine. In other words, countries in Eastern Europe are still very much dependent on one single source country (Russia) and one major transit country (Ukraine). Unexpectedly, we observe a spillover effect from countries, such as Germany, which make large investments in infrastructure. These countries themselves seem to benefit less from such investments than some of their smaller neighbours. The reason behind this spillover is that countries with plentiful access to network capacity provide routes for neighbouring countries to also access such capacity.

Figure 4A can be read from left to right: the scenarios that cause the largest disruption appear on the left, and the most benign scenarios are on the right. The present and future scenarios are clustered together when either Russia, Ukraine, the Netherlands, or Belarus are removed from the network, demonstrating that the new pipelines being built will only improve slightly the consequences of a hypothetical crisis with one of the major exporting countries (Russia or the Netherlands), or with a critical transit country (Ukraine or Belarus). It is thus very hard to change the consequences of such scenarios even by building new pipelines.

We illustrate our model at a fine geographical scale in the heat-map of Figure 4C, where we show the throughput for urban areas in Europe with 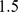 million inhabitants or more, as the scenarios vary. The figure suggests possible classifications of cities into groups, highlighted in gray. We observe in Figure 4D that the coefficient of variation is larger for cities in Eastern Europe than for cities elsewhere (except Berlin, Vienna and Dusseldorf). Note that Dublin is resilient to all scenarios because it is supplied from the UK, which we never removed from the network. Observe also that Austria gets most of its gas from Russia, and only a little from Norway, so Vienna is in a similar situation to Eastern European cities.

Taken together, Figures 4A–D illustrate the resilience of countries, urban areas and the network to the scenarios, by showing how countries and urban areas with similar reactions to different types of crises are grouped together by throughput or by its coefficient of variation, and how different scenarios are clustered by their effect on the countries and urban areas.

**Figure 5:**
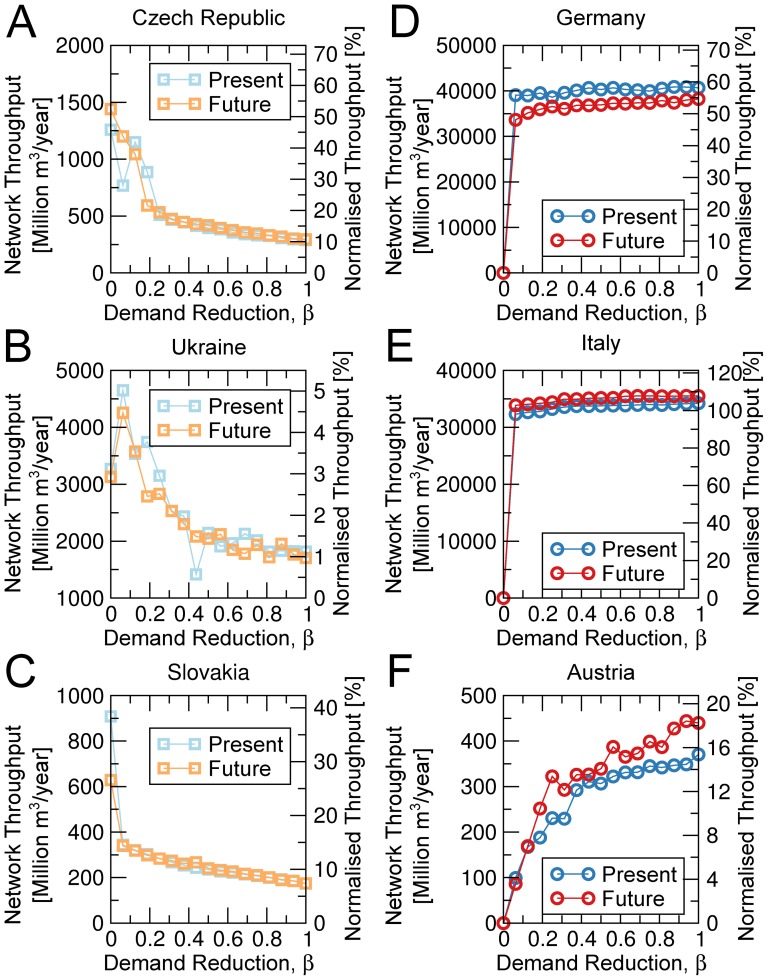
Network throughput of selected countries in a hypothetical crisis with Russia. The right axis shows the country throughput relative to the present baseline scenario. To minimize the impact of the loss of Russian supply, we re-allocate paths that originate in Russia to Norway and the Netherlands (see Methods). We then partition countries into two groups: group I is composed of Eastern Europe ( http://eurovoc.europa.eu/100277 ) together with Estonia, Finland, Greece, Latvia and Lithuania, and group II includes all other countries in our study. Group II countries have a demand of 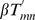, where 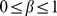. Panels (A)–(C) show the throughput for selected group I countries (open squares), whereas panels (D)–(F) illustrate the throughput for group II countries (open circles). Panels (A)–(C) demonstrate that countries in group I benefit from curtailing the demand of countries in group II. In contrast, panels (D)–(E) show that some countries in group II are largely unaffected even when their own demand is curtailed considerably. Finally, panel (F) demonstrates that supply to Austria is dominated by the demand reduction prefactor, 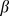. Indeed, Austria is crossed by routes from Norway and the Netherlands to group I countries, and these routes get a higher allocation of available capacity as Austrian demand decreases ( i.e., as 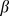 decreases).

The most challenging scenario is a hypothetical crisis that would cut-off supply from Russia to Europe. To investigate how Europe could make use of its internal gas production to minimize the impact of such a crisis, we simulate and quantify the effect of replacing gas supply from Russia with supply from Norway and the Netherlands. To do this, we start by creating a cut of European countries into two groups. Group I is made of the geographical cluster of countries that are heavily dependent on Russian gas, and is defined by Eastern Europe (http://eurovoc.europa.eu/100277) together with Estonia, Finland, Greece, Latvia and Lithuania. Group II is defined by all other countries in our study (see “Databases” in File S1). We consider a new scenario where Russia is removed from the network and the demand of countries in group I is rerouted to the Netherlands and Norway. To do this, we first create new paths linking each importing country in group I to Norway and the Netherlands (see “The Model” in File S1) and we update the matrix of gas flows to 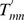 (see Methods). Next, we apply a prefactor 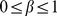 to the values of the demand 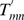 of countries in group II. The effect of 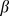 is to lower the utilization of the network by countries of group II that do not depend heavily on Russia. These countries typically have a high value of demand, and hence by curtailing their demand, there will be more capacity available to transport gas from Norway and the Netherlands to group I countries. In Figure 5, we observe that group I countries increase their access to network capacity as 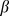 decreases. Group II countries, such as Austria, that are geographically on the main routes that link Norway and the Netherlands to group I countries, decrease their throughput as 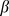 decreases. These countries are crucial: their throughput decreases as they share their network to benefit the more populous group I countries. In contrast, access to network capacity in routes supplying group II countries, such as Germany and Italy, is broadly unaffected, even as 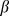 is lowered considerably, because routes from Norway and the Netherlands to group I countries use little network capacity from these group II countries. Despite the increase in throughput for countries in group I as 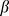 decreases, Figure 5 shows the difficulty in replacing Russia by the Netherlands and Norway. Although we can hope to recover between 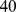 and 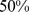 of the baseline throughput for the Czech Republic and Slovakia, we will only recover up to 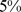 of the Russian supply to Ukraine and up to 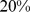 of the Austrian supply.

## Discussion

Agreed political management processes are needed for crises scenarios, to guarantee supply to the most affected countries and urban areas and minimize the loss of gas by populations. Here, we propose a decentralized algorithm inspired by congestion control on the Internet, which would eliminate the need of improvisation and complicated, lengthy negotiations every time a crisis occurs. Such mechanism has a stabilizing effect because it lowers the resource deficiency of the most affected countries [11], [27]. We demonstrate how a wide range of scenarios impacts network throughput at global, country and urban levels, and how countries and urban areas react to scenarios of hypothetical crises. We show and quantify how countries that are heavily dependent on Russian supply can lower the impact of a crisis, if other countries accept to reduce their demand. Finally, our model tries to systematically compare alternative policy options during energy crises, using complex system models [33].

In summary, Europe is not necessarily trapped and helpless during energy crises. The long-term interest in the sustainability of the gas industry makes governments and the industry likely to invest in rules and norms to enhance reciprocity and collective efforts during crises. Because the number of governments and companies ultimately involved in taking the decisions in Europe is relatively high, governments could implement decentralized solutions similar to the one we propose here, perhaps with a centralized control solution as backup. At its heart, energy security, like preparedness for future pandemics [34], is about cooperation among nations [1]. To avoid European-wide crises, nations must cooperate to share access to their critical infrastructure networks.

## Methods

Let 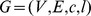 be an undirected and connected weighted graph with no loops, node-set 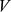 and link-set 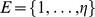. Each link 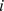 has a capacity 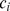 and a length 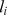. The network has a set of 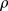 paths connecting source to sink nodes. All links of a path transport the same *path flow*. Different paths can share a link, even to perform transport in different directions ( e.g., , during distinct time intervals).

The relationship between links and paths can be described by the *link-path incidence matrix* 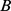 as follows. Set 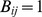 if the link 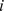 belongs to the path 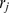, and set 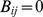 otherwise. Matrix 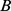 has dimensions 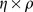, and maps paths to the links contained in these paths. When 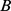 is applied to a vector of path flows, the resulting vector with components 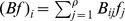 is the total flow on the links, or link throughput. We say that a link is a *bottleneck* if the sum of the path flows of paths that pass through it is equal to the link capacity. We assume that flows are elastic, that is path flows are not fixed by demand, but can be adjusted according to the available network capacity.

### Contract paths

The pipeline contracts are for physical point-to-point transport on a given system over a *contract path* [35], [36]. The contract path is a route between a pair of source and sink nodes, such that gas flows from source to sink along that path and the transport costs are only incurred on links along that route.

### Proportional fairness congestion control

The key to a decentralized algorithm for proportional fairness is to translate the optimization problem into an autonomous system of coupled differential equation, with a fixed point equivalent to the optimal solution of the optimization problem. To do this, we use the result that the stable fixed point of a system of differential equations is the maximum of the equations' Lyapunov function.

#### A primal algorithm

A decentralized algorithm for congestion control (see “Congestion Control” in File S1) solves the system of coupled ODEs:
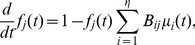
(1)where the price on link 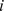 is
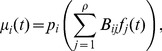
(2)and the price function is given by
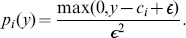
(3)


#### A dual algorithm

Consider a system where the shadow prices vary gradually as a function of the path flows (see “Congestion Control” in File S1):
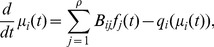
(4)where
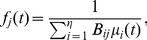
(5)and 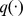 is the inverse of 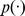. As 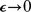, the dual and primal algorithms become equivalent. The rate of convergence to the stable point is a function of the link-path incidence matrix 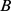 and of the derivatives of 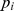 (primal algorithm) and 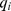 (dual algorithm), and increases with the magnitude of the latter [15].

### Rerouting the demand from Russia to the Netherlands and Norway

When Russia is removed from the network, we reroute paths between group I countries and Russia to paths between group I countries and the Netherlands and Norway. To do this, we pair the new source and sink nodes as described in Section “The Model” in File S1, but we modify the 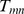 matrix of gas flows. The new 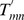 matrix is found by reallocating the demand from Russia for group I countries to the Netherlands and Norway, proportionally to the production of gas of these two exporting countries:
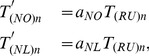
(6)where 
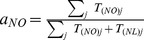
 and 
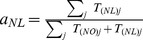
 are the normalised proportions of supply from Norway and the Netherlands, respectively.

## Supporting Information

File S1 **Supplementary information file which includes supplementary text, figures, and tables.**
(PDF)
