# Supplementary material for: Resilience of Natural Gas Networks during Conflicts, Crises and Disruptions
Source: PLoS One. 2014 Mar 12;9(3):e90265. doi: 10.1371/journal.pone.0090265 (PMC3951220; doi:10.1371/journal.pone.0090265)
Supplement: File S1 — Supplementary information file which includes supplementary text, figures, and tables. (PDF) [file pone.0090265.s001.pdf]

Supplementary Information: Resilience of natural gas networks during conflicts, crises and disruptions

Rui Carvalho[1,*], Lubos Buzna[2], Flavio Bono[3] Marcelo Masera[4] David K. Arrowsmith[1] Dirk Helbing[5,6]
1 School of Mathematical Sciences, Queen Mary University of London, Mile End Road, London E1 4NS, U.K.
2 University of Zilina, Univerzitna 8215/1, 01026 Zilina, Slovakia
3 European Laboratory for Structural Assessment, Institute for the Protection and Security of the Citizen (IPSC), Joint Research Centre, Via. E. Fermi, 2749 TP 480, Ispra 21027 (VA), Italy
4 Energy Security Unit, Institute for Energy and Transport, Joint Research Centre, Westerduinweg 3,NL-1755 LE Petten, The Netherlands
5 ETH Zurich, Clausiusstrasse 50, 8092 Zurich, Switzerland
6 Risk Center, ETH Zurich, Swiss Federal Institute of Technology, Scheuchzerstrasse 7, 8092 Zurich, Switzerland
∗ E-mail: r.carvalho@qmul.ac.uk

Contents

S1 Databases **2**
S1.1 First layer: population . . . 2
S1.2 Second layer: the gas pipeline network . . . 2
S1.3 Third layer: urban areas . . . 2
S1.4 Fourth layer: network of gas movements by pipeline and LNG . . . 2

S2 The Model **4**
S2.1 Tessellation of urban and non-urban areas and location of source and sink nodes . . . 4
S2.2 How we pair sink and source nodes . . . 4
S2.3 How we define demand . . . 4
S2.4 The problem with shortest path routing . . . 6
S2.5 How we determine the source to sink paths . . . 6

S3 Congestion Control **7**
S3.1 Proportional Fairness . . . 8
S3.2 A centralized algorithm for Proportional Fairness . . . 10
S3.3 A centralized algorithm for Proportional Fairness with link price . . . 11
S3.4 A decentralized algorithm for Proportional Fairness . . . 15

S4 Results **15**
S4.1 Detailed interpretation of results at country and urban levels . . . 15

S1 Databases

The data set is illustrated on Figures S1 and S2 and is the result of compiling GIS and population databases into several layers.

S1.1 First layer: population

We use the 2012 LandScan [1] high-resolution global population distribution data that estimates the population count with a spatial resolution of approximately 1 km, or 30 × 30 seconds of arc (see `http://www.ornl.gov/sci/landscan/`).

S1.2 Second layer: the gas pipeline network

We compiled the European gas pipeline transmission network and the Liquefied Natural Gas (LNG) terminals from the 2011 Platts Natural Gas geospatial data (see `http://www.platts.com/Products/gisdata`), including pipelines that are planned or under construction. The data set covers 25 of the 27 EU member states (except Malta and Cyprus), Belarus, Moldova, Western Russia, Ukraine (all part of the former USSR), Bosnia and Herzegovina, Croatia, Macedonia, Serbia (all part of the former Socialist Federal Republic of Yugoslavia), Algeria, Libya, Morocco, Tunisia (all part of the Maghreb), Norway, Switzerland and Western Turkey.

Similarly to electrical power grids, gas pipeline networks have two layers: transmission and distribution. The transmission network transports natural gas over long distances (typically across countries) and has a non-trivial topology. The distribution network is tree-like and comprises pipelines with smaller diameter that deliver gas to consumers. We extract the gas pipeline transmission network as all the important pipelines with diameter $d \geq 15$ inches. To finalize the network, we add pipelines interconnecting major branches, so that the resulting network is connected. Network links are weighted by pipeline diameter and length. To simplify, we assume that gas can flow on both directions of a pipeline, although over different time periods. The compiled network has $2,649$ nodes (compressor stations, city gate stations, Liquefied Natural Gas (LNG) terminals, etc.) connected by $3,673$ pipeline segments spanning $186,132$ km.

S1.3 Third layer: urban areas

To avoid the controversy in the definition of an urban area [2], we considered only urban areas with $100,000$ or more inhabitants as defined by the Eurostat urban audit (see `http://www.urbanaudit.org`). We are interested not just in the administrative boundaries of cities, but intend also to capture the surrounding areas that include a substantial share of the commuters into the city, since the gas pipeline infrastructure also supplies these peripheral urbanized districts. Note that the infrastructure network supplies directly the major urban areas, but may not intersect spatially with the built-up area of cities.

Urban areas in the European Union member countries and candidate countries are defined by Eurostat as *Larger Urban Zones* (`http://www.urbanaudit.org`), and the GIS files are provided by the European Environment Agency (`http://www.eea.europa.eu/data-and-maps/data/urban-atlas`). The city levels in non-EU countries are defined from remotely sensed data (see [3] and `http://www.naturalearthdata.com/downloads/10m-cultural-vectors/10m-urban-area/`). These city level areas are too small compared with the EU Larger Urban Zones. Hence, we define an urban area in non-EU countries to be the union of the third-level administrative divisions (`http://www.gadm.org/`) that intersect the corresponding city level polygon. We have found 376 urban areas with a total area of $723,957$ km^2.

S1.4 Fourth layer: network of gas movements by pipeline and LNG

The fourth layer is the network of annual movements of gas by pipeline and of Liquefied Natural Gas by ship into European terminals, collected from the International Energy Agency Natural Gas Information Statistics for 2011 [4] (see `http://www.oecd-ilibrary.org/statistics`). This directed network is represented by the weighted adjacency matrix $T_{mn}$of gas transported from m to n, where m stands either for a gas exporting country or for Liquefied Natural Gas (LNG) terminals that supply an importing country n (see Figure 2 of the main paper). We make use of ISO

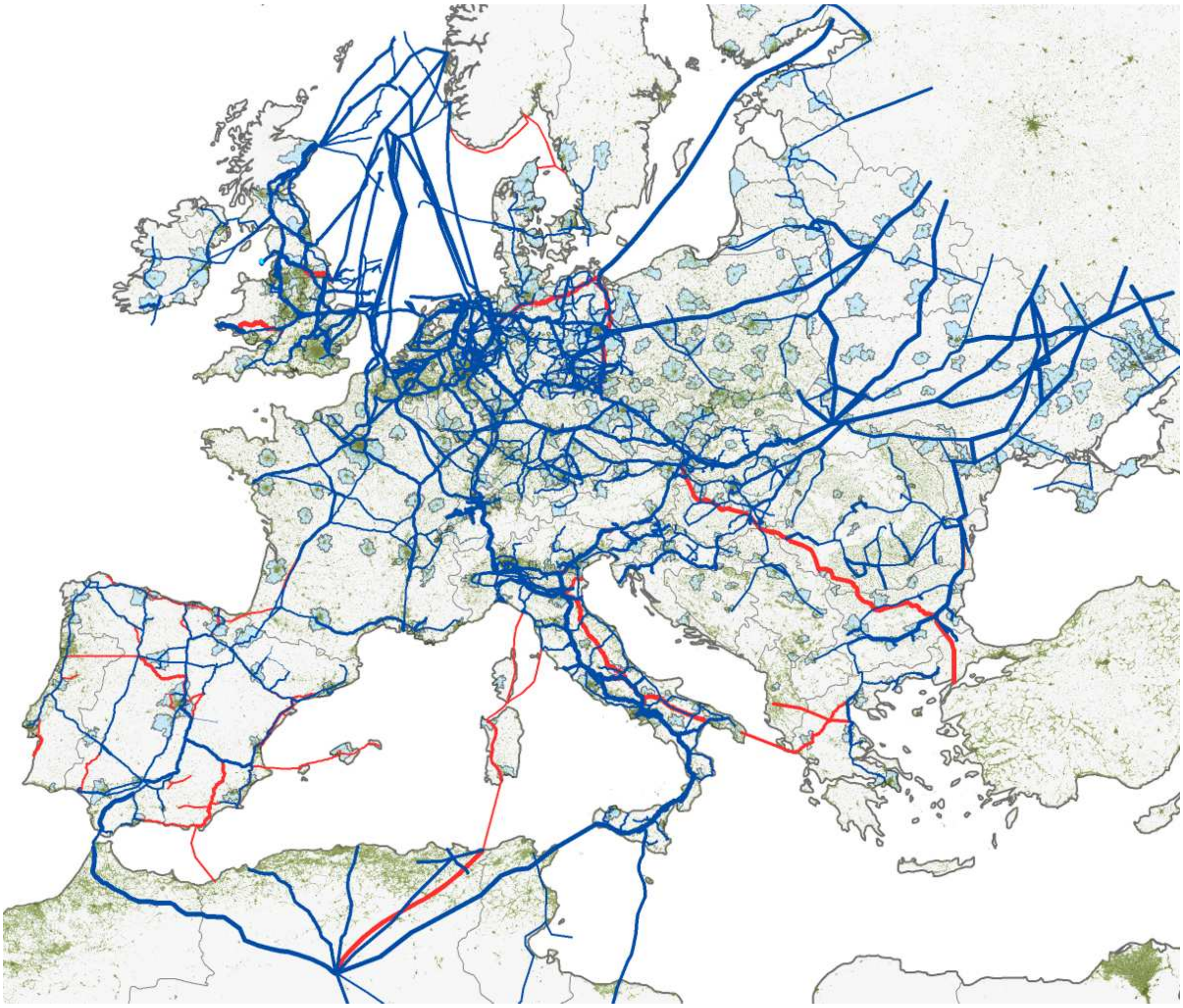

Figure S1. European gas pipeline network including part of North Africa. The present network is shown in dark blue, and the planned pipelines are shown in red. The population density is plot in dark green and Larger Urban Zones are indicated in cyan. Source: compiled from authors' data and Platts. Map composed in ESRI ArcGIS.

alpha-2 country codes in m and n to denote individual countries (see `http://www.iso.org/iso/home/standards/country_codes.htm`), so that, for example, $T_{(RU)(FR)}$ is the amount of gas imported annually by France from Russia.

S2 The Model

S2.1 Tessellation of urban and non-urban areas and location of source and sink nodes

For simplicity, we consider that all nodes in a gas exporting country are source nodes. The partition of non-urban areas is such that all points within a given Voronoi cell are closer to their corresponding gas pipeline node than to any other node. If a gas pipeline node is inside an urban polygon, we call it an urban node, otherwise, we say the node is non-urban. To simplify, we assume that an urban area is supplied by the pipeline links that cross its border and have a node inside its polygon. This node turns out to be also the urban node on the pipeline that is the closest to the border of the urban polygon, and is thus the first node that gas will cross along the pipeline when entering the urban area. Hence, we naturally say that the node is an urban sink, and we consider no other sink nodes along the pipeline for the given urban area. If an urban area polygon contains no gas nodes, we associate it to the closest gas node (urban or not) and say this node is a sink. We exclude pipeline links that have both end nodes located inside urban areas. In other words, we only consider pipeline links that have one urban and one non-urban node (see Figures S3 and S4).

S2.2 How we pair sink and source nodes

Source nodes are either located in an exporting country m, or at Liquefied Natural Gas (LNG) terminals. When m stands for a country, we connect by a path $r_{m,k,n,l}$ ($k = 1, \ldots, \Phi_{mn}$ and $l = 1, \ldots, t_n$) the t_n sinks in an importing country n to the Φ_{mn} closest source nodes in the exporting country m, where

$$\Phi_{mn} = \begin{cases} \min(10, s_m) & \text{if } m \text{ is a gas} \\ & \text{exporting country} \\ g_n & \text{if } m \text{ is LNG} \end{cases} \quad \text{(S1)}$$

In other words, when m is a country, we connect each sink node in an importing country n to a maximum of ten source nodes in an exporting country m. When m stands for LNG, we assume that sink nodes in an importing country n are supplied from all the LNG terminals in country n (see summary of the notation in Table 1). Since our congestion control algorithm shares available pipeline capacity, sink nodes can access at most the capacity available at cross-border links of exporting countries. We have a total of 15 nodes in Russia and 11 nodes in Norway, which link to cross-border pipelines and thus account for all the cross-border capacity between these exporting countries and all importing countries. Hence, our choice of a maximum $\Phi_{mn} = 10$.

S2.3 How we define demand

We define the demand of a country to be the amount of gas imported over the gas pipeline network and Liquefied Natural Gas terminals, as given by the T_{mn} matrix (see Figure 2 of the main paper), and the demand of a given geographical area to be the demand of the country weighted by the ratio between the area and the country populations. Since demand for energy is proportional to population [5], we locate the sink nodes and associate them with the population they supply. When the area is urban, we split its total population equally among the sink nodes inside the urban polygon. If an urban area contains no gas nodes inside its polygon, we add its population to the population associated with the closest gas node. In non-urban areas, we associate the gas pipeline node at centre of a Voronoi cell with the population of the cell. Because each sink node in an importing country n is connected by Φ_{mn} paths to source nodes in an exporting country m, each of these paths has a share of the demand T_{mn} given by

$$D_{mnl} = \frac{1}{\Phi_{mn}} \frac{Z_{nl} T_{mn}}{z_n} \quad \text{(S2)}$$

where Z_{nl} is the population associated with sink node l of importing country n, z_n is the population of importing country n, T_{mn} is the volume of gas imported by an importing country n from an exporting country m, and the number Φ_{mn} of paths from an exporting country m to each sink node is given by equation (S1) (see summary of the notation in Table 1).

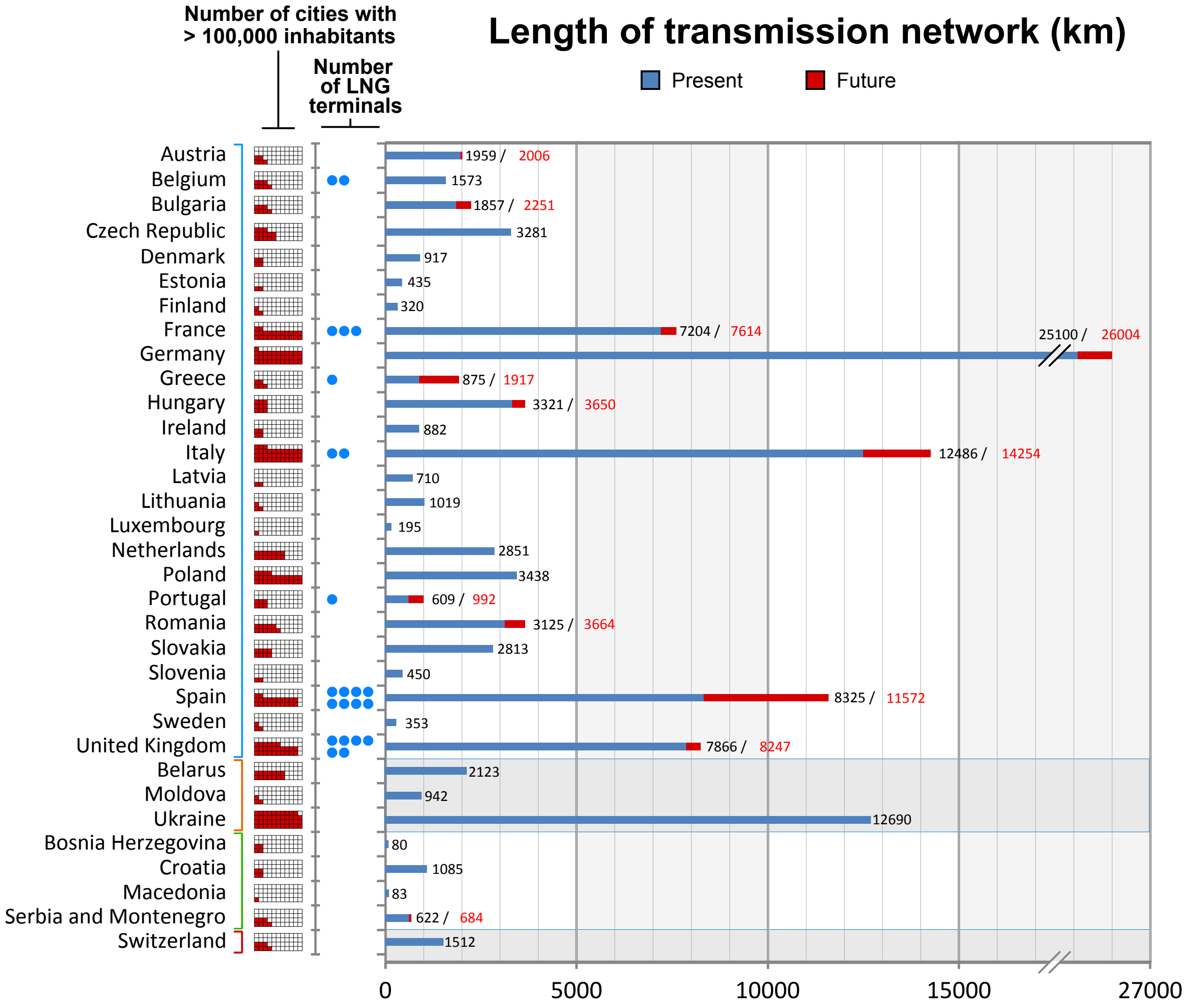

Figure S2. Number of urban areas and Liquefied Natural Gas terminals, and length of the present and planned gas pipeline networks of the countries analysed.

We next express the demand of a path in units of the minimum demand on the network. To do this, we note that D_{mnl} is independent of k, and we replace path $r_{m,k,n,l}$ having demand D_{mnl} by $\overline{D}_{mnl}$ paths identical to $r_{m,k,n,l}$, each with demand $\min(D_{mnl})$, where

$$\overline{D}_{mnl} = \left\lfloor \frac{D_{mnl}}{\min(D_{mnl})} \right\rfloor, \tag{S3}$$

where $\lfloor\cdot\rfloor$ is the largest integer not greater than $\cdot$. We note that the demand of a sink node from a source node is now proportional to the number of paths connecting the source and sink pair.

The path notation $r_{m,k,n,l}$ has been useful so far to locate the origin and destination of the paths, but the congestion control algorithm uses matrix multiplication, and it is simpler from now on to index paths in the network by an integer. To do this, we loop through all pairs of exporting and importing countries with a non-zero entry in the T matrix and re-label each of the $\overline{D}_{mnl}$ source-sink paths identical to $r_{m,k,n,l}$ by the new index. In other words, for each pair of importing-exporting countries, we go through the $\overline{D}_{mnl}\Phi_{mn}t_n$ shortest paths that connect source to sink nodes (see Table 1), and we index all paths in increasing order of first m, then n and finally k. Now that we have allocated the source to sink paths, we update the number of paths on the network $\rho = \sum_{i=1}^{\nu}\sum_{j=1}^{\nu}\sum_{l=1}^{t_n}\overline{D}_{mnl}\Phi_{mn}\widehat{T}_{mn}$, where $\widehat{T}_{mn} = 1$ if T_{mn} is positive and zero otherwise, and we write r_j to denote path j, where $j = 1, \ldots, \rho$.

S2.4 The problem with shortest path routing

The pattern of route intersection determines how much the paths condition each other in their sharing of network links, and the capacity of links limits how much can be transported locally. If the network is not congested, transport over the geographical shortest paths minimizes the costs. In contrast, shortest path routing in congested networks can be inefficient, because it may cause congestion at a few overloaded links, while avoiding alternative routes that are only slightly longer but have higher capacity. Moreover, routing over shortest paths in gas pipeline networks makes the effect of congestion even worst. Indeed, parallel routes with similar capacity are often available, but only one of these routes is the shortest path (see Figure S5), and hence the network capacity is largely underused.

S2.5 How we determine the source to sink paths

To begin integrating routing and congestion control, we consider first how to distribute the capacity c_i of a congested link i over the $1 + b_i = 1 + \sum_{j=1}^{\rho} B_{ij}$ paths that pass through the link when we add a new path through i, where B is the link-path incidence matrix ($B_{ij} = 1$ if the link i belongs to the path r_j and $B_{ij} = 0$ otherwise). An equitable way to divide the capacity on the link is to assign a path flow of $h_i = c_i/(1 + b_i)$ to each of the $1 + b_i$ paths. Intuitively, h_i is the slice of capacity allocated in a fair way to each of the b_i paths that share the capacity c_i, and the split is viewed as a fair outcome [6]. Moreover, $1/h_i$ can be interpreted as a simple measure of network congestion, since it has a maximum at the most congested link [7]. Hence, we combine routing and congestion through an effective link length:

$$\widetilde{l}_i = \left(\frac{\langle h_i\rangle}{h_i}\right)^{\alpha} l_i, \tag{S4}$$

where $\langle h_i\rangle$ is the average of h_i over all network links, l_i is the length of link i, and $0 \leq \alpha < 1$. Whereas we weight links by their length l_i in the calculation of geographical shortest paths, we now weight each link by $\widetilde{l}_i$ in the calculation of weighted shortest paths. Thus, a link becomes less attractive (its effective length is increased) if it is more congested than the average. We find that the global network throughput is maximized for $\alpha = 0.03$ (see Figure S6), and thus we use this value in the simulations.

We define the effective path length $\overleftrightarrow{l_j}$ of path j as the sum of the effective lengths of each of its links. We interpret the sum of link weights on a path as a penalty, which we then use to reroute paths iteratively via the following heuristic [8]. We i) go through each source and sink node pair and find a new path j connecting the two nodes; ii) if this new path has lower value of $\overleftrightarrow{l_j}$ than the previously found path, then it replaces the existing source to sink path; iii) we recompute the weights $\widetilde{l}_i$ for all

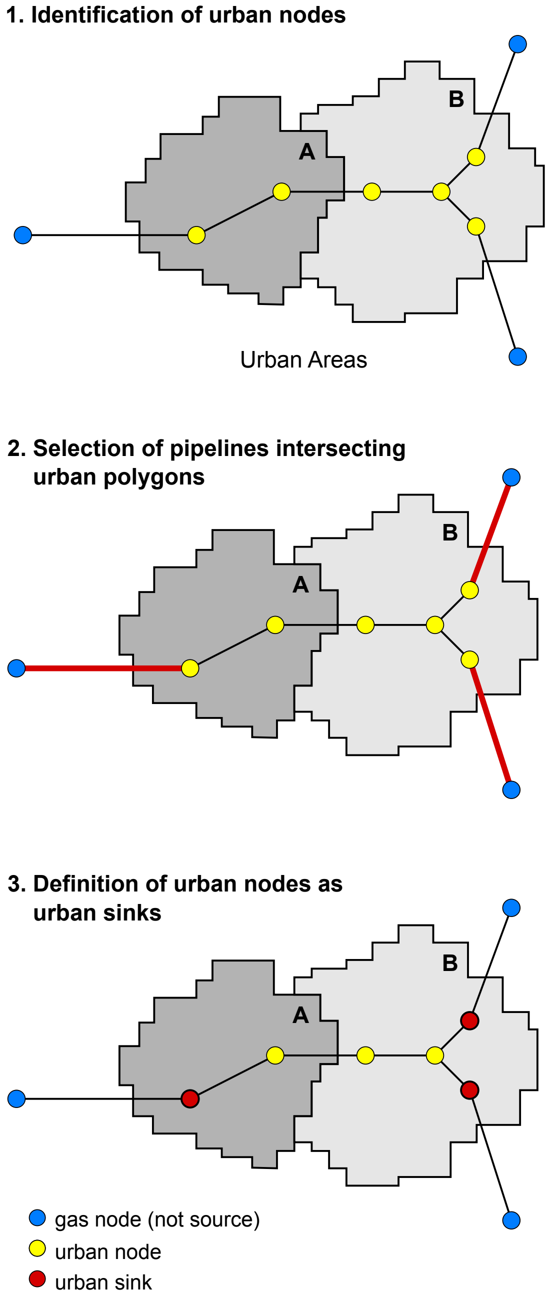

Figure S3. Schematic figure illustrating the allocation of sink nodes. Urban sink nodes are shown in red, urban nodes that are not sinks are shown in yellow, and non-urban sink nodes are shown in blue.

links on the new paths and repeat the procedure for all paths, until it has been executed 20 times (we found that the solution is does not change significantly when the number of iterations is larger than 20).

S3 Congestion Control

How should we allocate scarce network resources to competing paths so as to manage network congestion? There are two mechanisms at play in such allocation. On one hand, maximizing the flow transported on the network may lead to some paths being assigned a zero share of network capacity, and hence zero path flow. These paths are effectively blocked from using the network, and hence the flow allocation is unfair. On the other hand, allocations that share network capacity fairly are known to deliver low throughput and are thus inefficient [7]. Hence, a good solution to the problem of congestion control aims at a trade-off between efficiency and fairness.

How should we generalize equation (S4) when paths pass through several congested links on the network? Our intuitive notion of fairness breaks down on networks, because paths typically cross several congested links and hence share the capacity of these links with other paths. Roughly, a solution is to allocate path flows iteratively, such that at each iteration we increase all path flows that do not pass through existing bottleneck links by the slice of capacity that is found by sharing equitably the capacity of the most congested links. The slice of capacity available to each path is the smallest on the most congested links, that is, the ratio h is the smallest on these links. A procedure to do this finds one link i, with the smallest ratio h_i and increases all path flows by h_i. Such procedure distributes parsimoniously the capacity of link i among the paths that pass through the link, and increases all unsaturated path flows by h_i. The procedure then fixes the path flows of the paths that cross links with capacity c_i, and decreases the capacity of links crossed by these paths by the amount of flow fixed [1]. This creates a residual network, on which the procedure is then repeated, such that path flows are saturated and the capacity available at the links they cross is updated at each iteration. The procedure is re-

[1] In general, there may be more than one link with minimum h_i, in which case all of such links are saturated.

peated until all paths in the network are saturated. Such allocation is known as *max-min fair* [9, 7], a name that comes from the way that path flows with minimum allocation are maximized by splitting equitably the capacity at the bottleneck links in an iterative process. The max-min fair allocation is such that to increase a path flow we have to decrease another path flow that is already smaller.

The major limitation of the max-min fair method is that network throughput is low compared to max-flow. To understand the mechanism behind this, we have to look at how both max-min fair and max-flow allocate path flows. The efficient allocation (max-flow) privileges short, over long paths that pass through several bottleneck links. Long paths take up capacity from other paths at each bottleneck, but only contribute to network throughput at the sink node. Hence, network throughput is maximized by minimizing the share of capacity to the long paths that pass through many bottlenecks, so that shorter paths can get a higher allocation of capacity and thus provide a higher contribution to network throughput. On the other hand, the max-min fair allocation shares the capacity of bottlenecks among the paths that pass through them. Thus, unlike max-flow, max-min fair allocations do not restrict the amount of network capacity that long paths can consume and are often inefficient. This limitation prompted the search for a trade-off between max-min fairness and max-flow, which would still distribute network capacity in an equitable way, and thus *proportional fairness* appeared in the late 1990s.

S3.1 Proportional Fairness

Both proportional fairness and max-min fairness share the capacity c_i of a single link among N paths in a fair way, so that each path gets a path flow of c_i/N, but the two allocations are distinct when operating on a network.

Definition 1 *A vector of path flows $f^* = (f_1^*, \ldots, f_\rho^*)$ is proportionally fair if it is feasible and if for any other feasible vector of path flows f, the sum of proportional changes in the path flows is non-positive [10, 11]:*

$$\sum_{j=1}^{\rho} \frac{f_j - f_j^*}{f_j^*} \leqslant 0. \qquad \text{(S5)}$$

If there were no capacity constraints, equation (S5) would be verified when $f_j^* = \infty$ for all $j = 1, \ldots, \rho$. The capacity constraints imply that a flow allocation f^* is proportionally fair if all other feasible vector of path flows $f_j = (1 + \delta_j) f_j^*$, for $\delta \in \mathbb{R}^\rho$ where $j = 1, \ldots, \rho$, verify that the aggregate of percent changes $\sum_{j=1}^{\rho} \delta_j$ is non-positive.

Theorem 1 *The unique set of feasible paths flows that maximizes the function $U(f) = \sum_{j=1}^{\rho} \log(f_j)$ is proportionally fair.*

Proof. The proof given here is a direct application of the properties of convex functions [12, 13] and global maxima of a function (a sketch of the proof is given in [14]). First, observe that the set of feasible path flows is compact (closed and bounded) and convex. The functions $\log(f_j)$ are strictly concave, and thus $U(f)$ is strictly concave, since it is the sum of strictly concave functions. Thus $U(f)$ has a unique global maximum. Second, note that the tangent plane at any point of a convex (concave) function lies below (above) the graph of the function. Hence, since $U(f)$ is concave:

$$\nabla U(f) \cdot (g - f) \geqslant U(g) - U(f). \qquad \text{(S6)}$$

Now assume that f is a proportionally fair allocation. Then, $\nabla U(f) \cdot (g - f) \leqslant 0$ from equation (S5), and thus $U(f) - U(g) \geqslant 0$ from equation (S6) for all other feasible g. Hence, f is a global maximum of U. Conversely, assume that the function U has one global maximum at $U(f)$. Then,

$$\nabla U(f) \cdot (g - f) = \lim_{t \to 0^+} \frac{U(f + t(g - f)) - U(f)}{t} \leq 0,$$

and thus the flow allocation is proportionally fair. □

Theorem 2 *If a vector $f^* = (f_1, \ldots, f_\rho)$ of path flows is proportionally fair, then each path will pass through a bottleneck.*

Proof. To see this, assume that there is one path r_j that does not pass through any bottleneck. Consider link $i \in E(r_j)$ on the path r_j. The path flow f_j can be increased by $\delta = \min_{i \in E(r_j)} \{c_i - \sum_{k=1}^{\rho} B_{i,k} f_k\} > 0$, such that the new vector of path flows is $f' = (f_1, \ldots, f_j + \delta, \ldots, f_\rho)$. Hence, f' is not proportionally fair because $\sum_{q=1}^{\rho} (f'_q - f_q)/f_q = \delta / f_j > 0$, and the path flow f_j can be increased. □

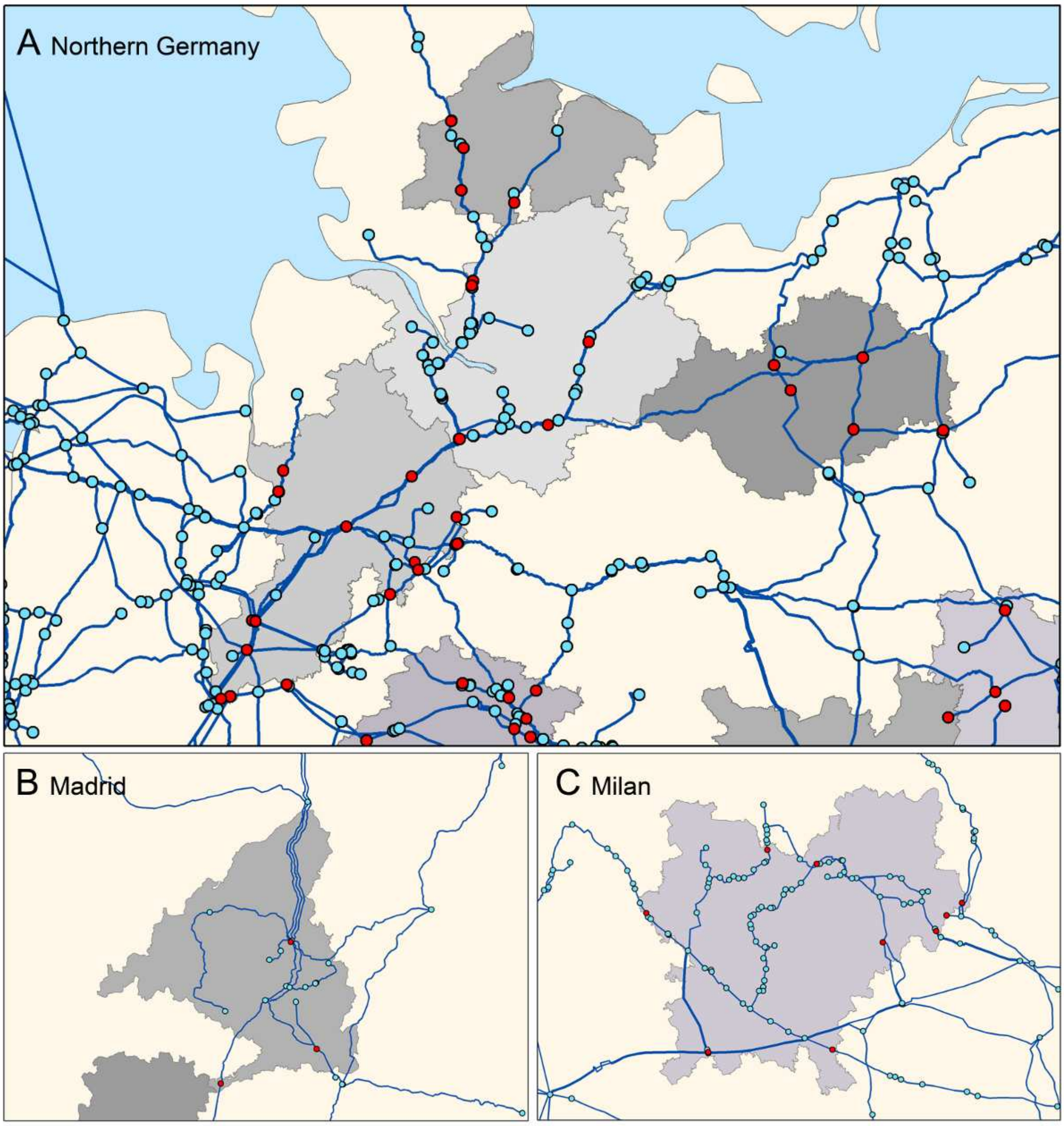

Figure S4. Urban sink nodes are highlighted in red for the Larger Urban Zones of (A) Hamburg and North West Germany, (B) Madrid, and (C) Milan. Source: compiled from authors' data and Platts. Map composed in ESRI ArcGIS.

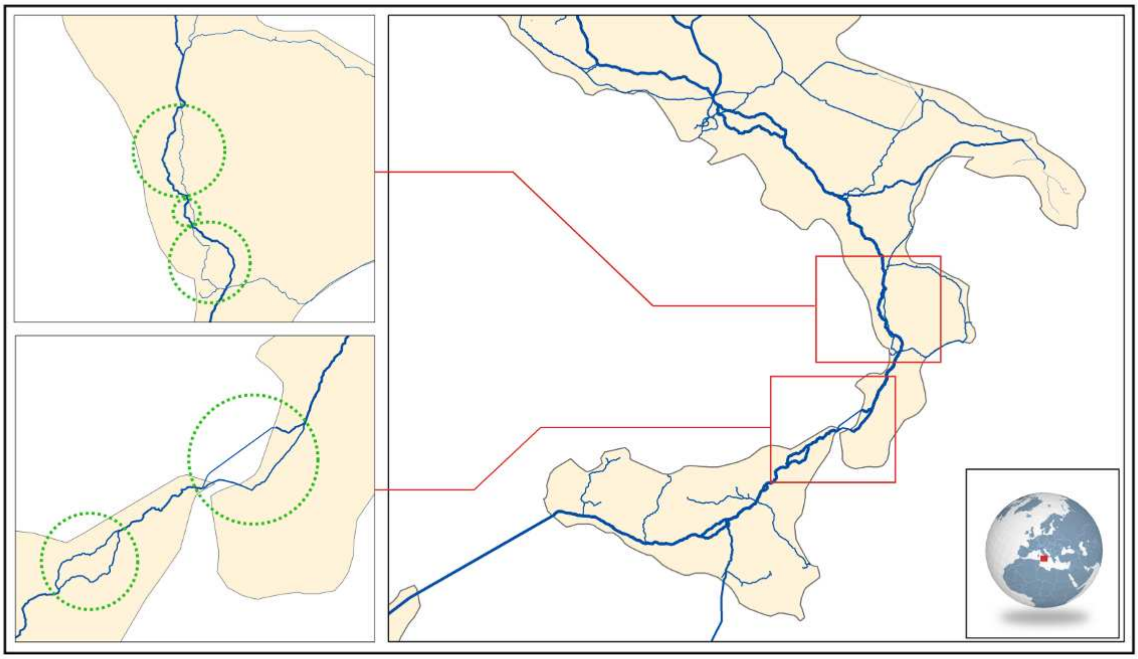

Figure S5. Detail of the gas pipeline network in Italy, showing the presence of parallel routes. Source: compiled from authors' data and Platts. Map composed in ESRI ArcGIS.

S3.2 A centralized algorithm for Proportional Fairness

Now in order to find the proportionally fair allocation, we need to maximize $U(f)$, constrained to the vector of path flows being feasible, that is:

$$\begin{aligned} \underset{f}{\text{maximize}} \quad & U(f) = \sum_{j=1}^{\rho} \log(f_j) \\ \text{subject to} \quad & Bf \leq c \\ & f_j \geq 0, \end{aligned} \tag{S7}$$

where the link-path incidence matrix is defined by $B_{ij} = 1$ if the link i belongs to the path r_j and $B_{ij} = 0$ otherwise, and $c = (c_1, \ldots, c_\eta)$ is the vector of link capacities. The aggregate utility $U(f)$ is concave and the inequality constraints are convex, and hence the optimization problem (S7) is convex. Thus, any locally optimal point is also a global optimum and we can use results from the theory of convex optimization to solve problem (S7) (see [15] and [16] for a brief introduction to Lagrange multipliers, and [17] on convex optimization). The

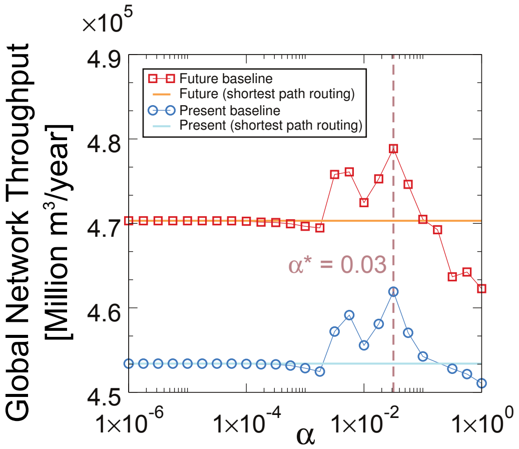

Figure S6. Plot of the global network throughput in the present and future baseline scenarios when we apply the heuristic routing algorithm of Equation (S4). Horizontal lines are a guide for the eye and show network throughput in the present and future baseline scenarios with shortest path routing. We choose the value $\alpha = 0.03$ that maximizes the global throughput.

Lagrangian associated with the optimization problem (S7) is [10, 11]:

$$L(f,\mu) = \sum_{j=1}^{\rho} \log(f_j) + \mu^T(c - Bf) \qquad \text{(S8)}$$

where $\mu = (\mu_1, \ldots, \mu_\eta)$ is a vector of Lagrange multipliers. The Lagrange dual function [17] is then given by $\sup_f L(f,\mu)$, which is easily determined analytically by $\partial L(f^*,\mu^*)/\partial f = 0$ as

$$\frac{\partial L(f^*,\mu^*)}{\partial f_j^*} = \frac{1}{f_j^*} - \sum_{i=1}^{\eta} B_{ij}\mu_i^* = 0 \Leftrightarrow$$
$$f_j^* = \frac{1}{\sum_{i=1}^{\eta} B_{ij}\mu_i^*}, \qquad \text{(S9)}$$

and thus

$$\sup_f L(f,\mu) = -\sum_{j=1}^{\rho} \log\left(\sum_{i=1}^{\eta} B_{ij}\mu_i\right) + \sum_{i=1}^{\eta} \mu_i c_i - \rho \qquad \text{(S10)}$$

After removing the constant term in equation (S10) and converting to a maximization problem, we obtain the dual problem [10, 11]

$$\begin{aligned} &\underset{L}{\text{maximize}} && V(\mu) = \sum_{j=1}^{\rho} \log\left(\sum_{i=1}^{\eta} B_{ij}\mu_i\right) - \sum_{i=1}^{\eta} \mu_i c_i \\ &\text{subject to} && \mu_i \geq 0. \end{aligned} \qquad \text{(S11)}$$

The primal problem (S7) is convex and the inequality constraints are affine. Hence, Slater's condition is verified and thus strong duality holds. This means that the *duality gap*, i.e., the difference between the optimal of the primal problem (S7) and the optimal of the dual problem (S11), is zero [17]. Strong duality has potentially immense implications as, depending on the problem, it may be easier to solve the primal or the dual. In our case, the primal objective function depends on ρ variables (the path flows) and is constrained by an affine system of equations, whereas the dual objective function depends on η variables (the links) and is constrained only by the condition that the Lagrange multipliers are non-negative. Taken together, the methods of convex optimization provide us with powerful tools to gain insights into patterns of congestion in networks where the number ρ of transport routes can be considerably larger than the number η of available transport links. Strong duality then states that the optimal path flows f^* are related to the optimal Lagrange multipliers μ^* by equation (S9).

Since f^* maximizes the Lagrangian over f, it follows that its gradient must vanish at f^*, and thus the following *KKT* condition is satisfied:

$$\mu_i^* \left(c_i - \sum_{j=1}^{\rho} B_{ij} f_j^* \right) = 0. \qquad \text{(S12)}$$

Equation (S12), often referred to as *complementary slackness* [17], states that the vector μ of the Lagrange multipliers and the vector of residual capacity have complementary sparsity patterns. To be more specific, either link i is utilized to full capacity (i.e., $c_i = \sum_{j=1}^{\rho} B_{ij} f_j^*$) and $\mu_i^* > 0$, or $\mu_i^* = 0$ and the capacity of link i is underused (i.e., $\sum_{j=1}^{\rho} B_{ij} f_j^* < c_i$). This gives us a simple and powerful way to identify bottleneck links numerically, as the links with a positive Lagrange multiplier μ_i^*.

S3.3 A centralized algorithm for Proportional Fairness with link price

Now suppose that the network operator charges a price per unit flow $p_i(y)$ for the use of link i, when the total load on the link is $y = \sum_{j=1}^{\rho} B_{ij} f_j$. This means that the price at one link depends on all the paths that pass through the link [11]. Hence, the problem (S7) can be generalized by adding a cost or penalty that is a function of the price [10, 11]. If the penalty is infinite when the link capacity is exceeded, $y > c_i$, then we can generalize problem (S7) to replace the capacity constraints by the link cost, such that

$$\begin{aligned} &\underset{f}{\text{maximize}} \\ &\widehat{U}(f,p,y) = \sum_{j=1}^{\rho} \log(f_j) - \sum_{i=1}^{\eta} \int_0^{y_i} p_i(z)dz \qquad \text{(S13)} \\ &\text{subject to} \\ &Bf = y \\ &f_j, y_i \geq 0. \end{aligned}$$

To derive the dual of problem (S13), we first find

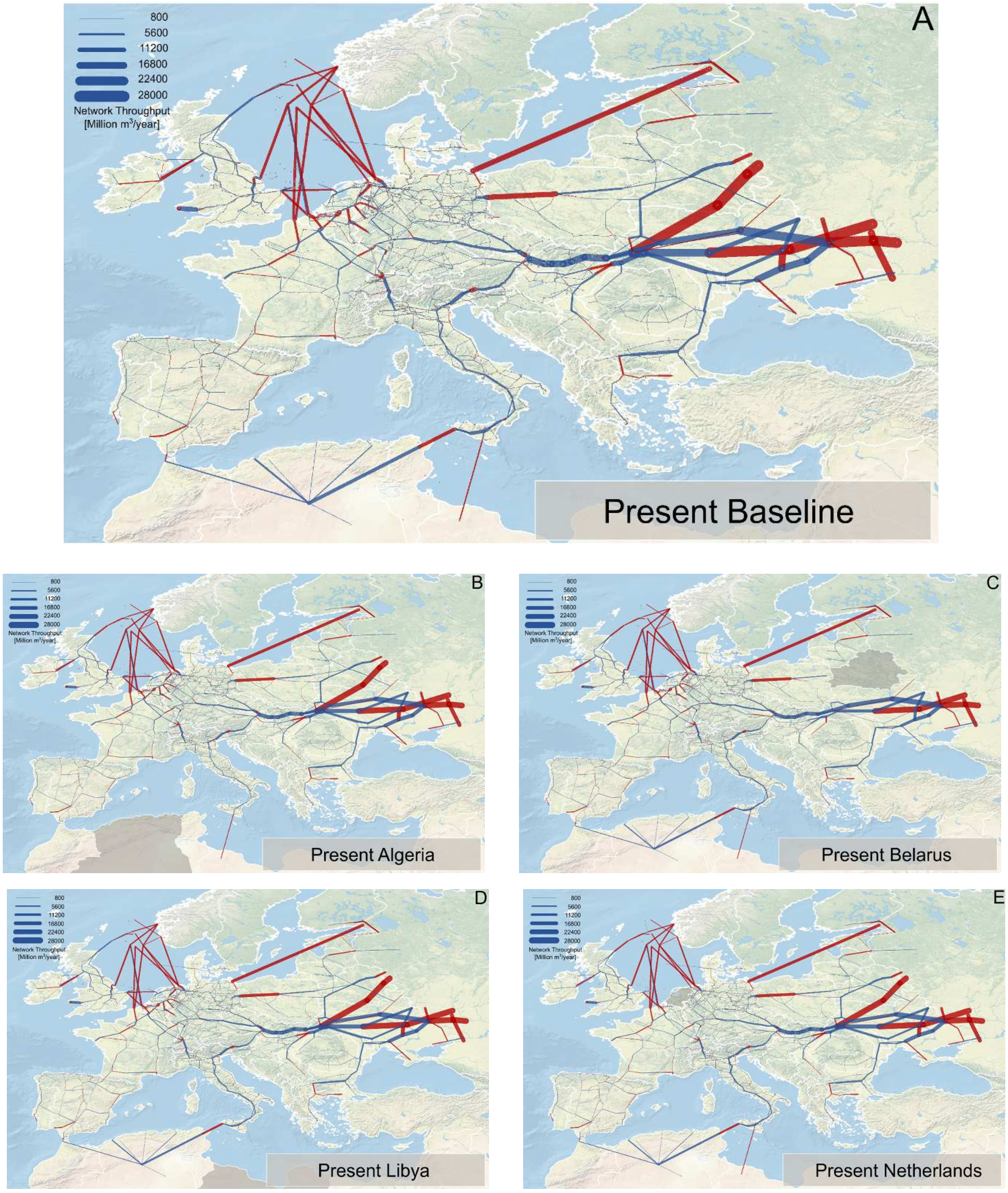

Figure S7. Map of the flow allocation in the following scenarios: (A) present network, (B) present network without Algeria, (C) present network without Belarus, (D) present network without Libya, and (E) present network without the Netherlands. Link thickness is proportional to the total flow on the link. Links in dark red are bottlenecks and links in blue are not used to their full capacity. The scenario country, which is hypothetically removed from the network, is highlighted in gray on the map. Source: compiled from authors' data and Platts. Map composed in ESRI ArcGIS.

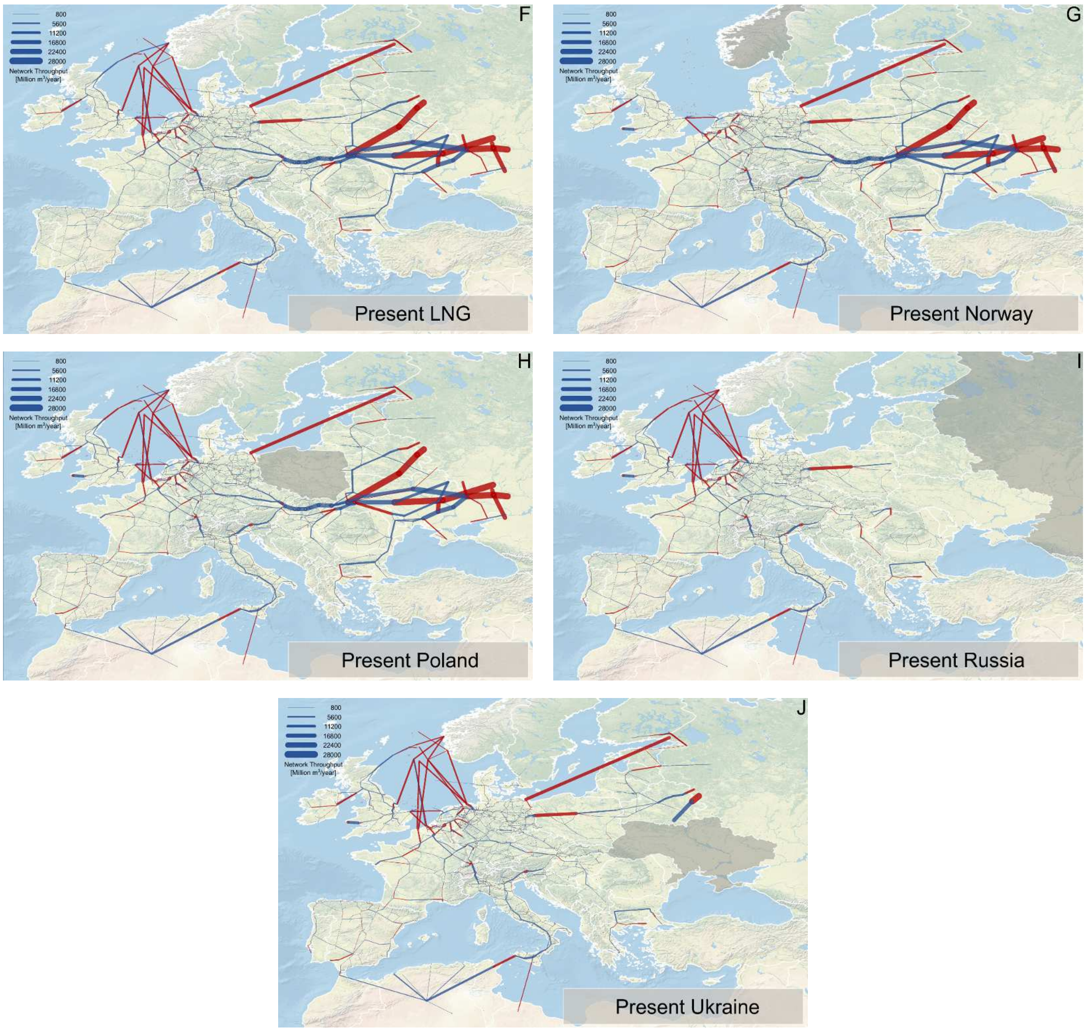

Figure S8. Map of the flow allocation in the following scenarios: (F) present network without Liquefied Natural Gas (LNG) terminals, (G) present network without Norway, (H) present network without Poland, (I) present network without Russia, (J) present network without Ukraine. Link thickness is proportional to the total flow on the link. Links in dark red are bottlenecks and links in blue are not used to their full capacity. The scenario country, which is hypothetically removed from the network, is highlighted in gray on the map. Source: compiled from authors' data and Platts. Map composed in ESRI ArcGIS.

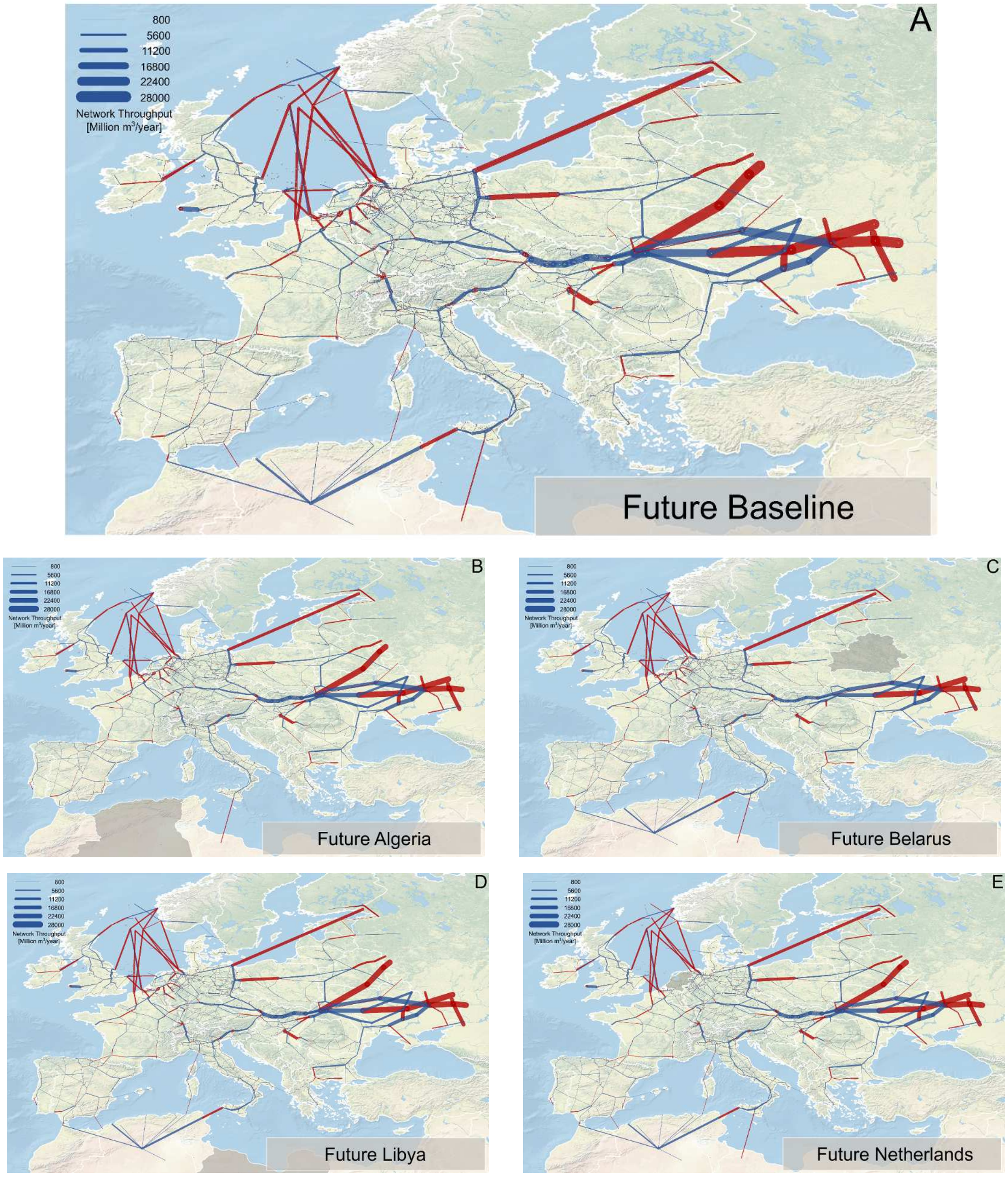

Figure S9. Map of the flow allocation in the following scenarios: (A) future network, (B) future network without Algeria, (C) future network without Belarus, (D) future network without Libya, and (E) future network without the Netherlands. Link thickness is proportional to the total flow on the link. Links in dark red are bottlenecks and links in blue are not used to their full capacity. The scenario country, which is hypothetically removed from the network, is highlighted in gray on the map. Source: compiled from authors' data and Platts. Map composed in ESRI ArcGIS.

its Lagrange dual

$$\widehat{g}(\mu) = \sum_{j=1}^{\rho} \log\left(\frac{1}{\sum_{i=1}^{\eta} B_{ij}\mu_i}\right) - \rho + \sum_{i=1}^{\eta}\left(\mu_i p_i^{-1}(\mu_i) - \int_0^{p_i^{-1}(\mu_i)} p_i(z)dz\right) \quad \text{(S14)}$$

To simplify equation (S14), we integrate by parts and then by substitution,

$$\widehat{g}(\mu) = \sum_{j=1}^{\rho} \log\left(\frac{1}{\sum_{i=1}^{\eta} B_{ij}\mu_i}\right) - \rho + \sum_{i=1}^{\eta} \int_0^{\mu_i} q_i(x)dx, \quad \text{(S15)}$$

where $q(\cdot)$ is the inverse of $p(\cdot)$. Following [10, 11], we now remove the constant term in equation (S15) and covert to a maximization problem to obtain the dual of problem (S13):

$$\begin{aligned} &\underset{L}{\text{maximize}} \\ &\widehat{V}(\mu, q) = \sum_{j=1}^{\rho} \log\left(\sum_{i=1}^{\eta} B_{ij}\mu_i\right) - \sum_{i=1}^{\eta} \int_0^{\mu_i} q_i(x)dx \\ &\text{subject to} \quad \mu_i \geq 0. \end{aligned} \quad \text{(S16)}$$

The dual problem (S16) is equivalent to the original dual problem (S11) if $q_i(x) = c_i$. However, this function is non-invertible and thus we approximate it by the invertible function [10, 11]

$$q_i(x) = \frac{xc_i}{x+\epsilon}. \quad \text{(S17)}$$

Problems (S16) and (S11) are thus equivalent in the limit $\epsilon \to 0$. The primal problems (S7) and (S13) are equivalent in the limit $\epsilon \to 0$ if

$$p_i(y) \sim q_i^{-1}(y) = y\epsilon/(c_i - y). \quad \text{(S18)}$$

S3.4 A decentralized algorithm for Proportional Fairness

For each path r_j, the network is offering a certain path flow f_j with unit rate of change, $df_j/dt = 1$. Now suppose that the network operator charges a price per unit flow $p_i(y)$ for the use of link i, when the total load on the link is $y = \sum_{j=1}^{\rho} B_{ij} f_j$. This means that the price at one link depends on all the paths that pass through the link. The price causes a reduction in the path flow f_j, such that

$$\frac{d}{dt} f_j(t) = 1 - f_j(t) \sum_{i=1}^{\eta} B_{ij}\mu_i(t), \quad \text{(S19)}$$

where the price on link i is

$$\mu_i(t) = p_i\left(\sum_{j=1}^{\rho} B_{ij} f_j(t)\right). \quad \text{(S20)}$$

User i responds to an underused capacity with a steady increase of its path flow, and to congestion with a multiplicative decrease of its path flow at a rate proportional to the congestion price. This additive-increase/multiplicative-decrease mechanism is best known for its use in communication networks, and is implemented in TCP congestion avoidance [18, 19].
A possible pricing policy consists in charging only for link flows that are close to capacity, with a sharp increase in the price that each path pays as the link becomes saturated:

$$p_i(y) = \frac{\max(0, y - c_i + \epsilon)}{\epsilon^2}. \quad \text{(S21)}$$

As $\epsilon \to 0$, the price p_i tends to zero for link flows below capacity, and to infinity for saturated links. Hence, problem (S13) approximates arbitrarily closely the primal problem (S7).

S4 Results

Figures S7, S8, S9 and S10 show the load on network links in the present and future scenarios. These figures demonstrate that our model reproduces the main transport corridors in Europe, and show the spatial pattern of bottleneck links for each scenario. It is apparent how a hypothetical removal of either Russia or Ukraine cuts-off the major transport routes, and damages drastically the supply of populations in Europe.

S4.1 Detailed interpretation of results at country and urban levels

At country level:

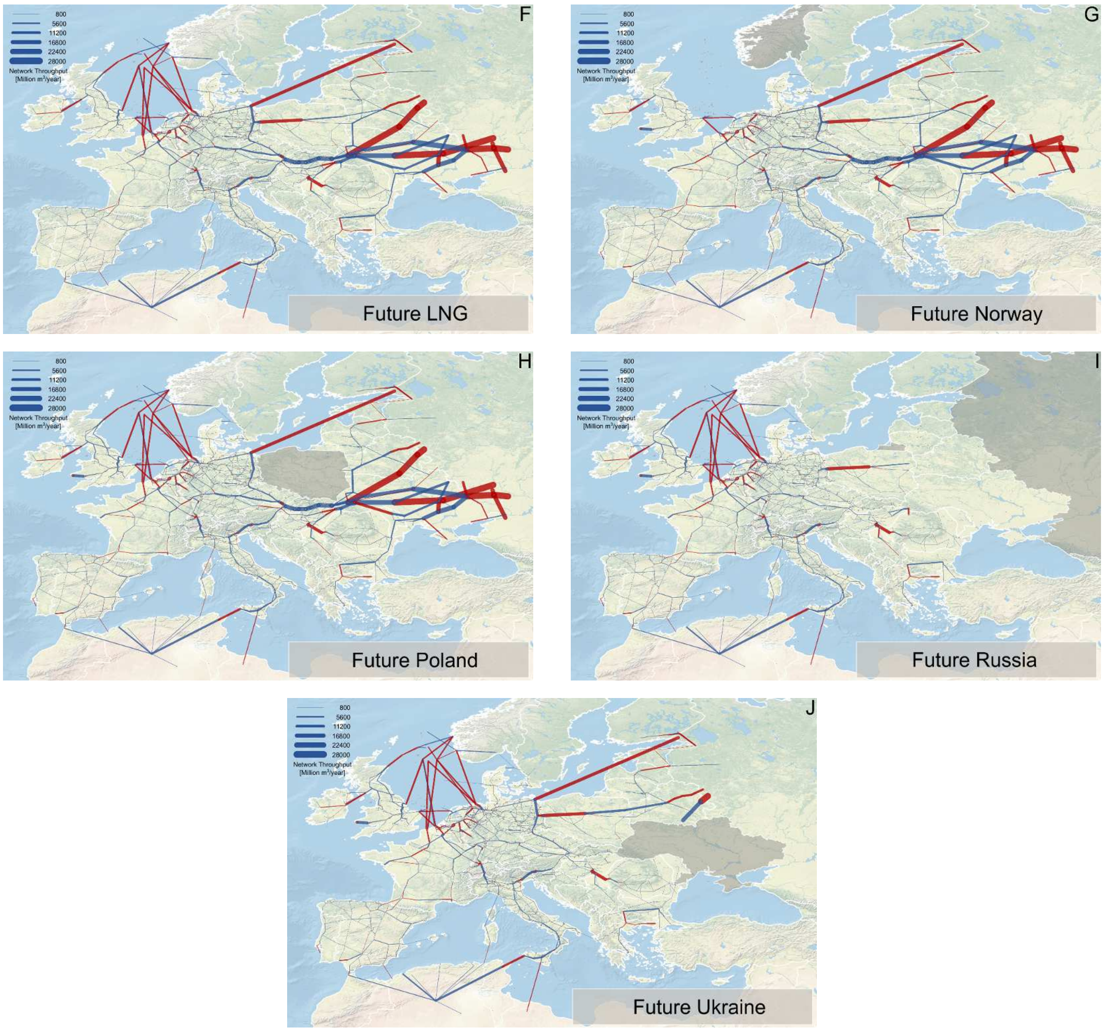

Figure S10. Map of the flow allocation in the following scenarios: (F) future network without Liquefied Natural Gas (LNG) terminals, (G) future network without Norway, (H) future network without Poland, (I) future network without Russia, (J) future network without Ukraine. Link thickness is proportional to the total flow on the link. Links in dark red are bottlenecks and links in blue are not used to their full capacity. The scenario country, which is hypothetically removed from the network, is highlighted in gray on the map. Source: compiled from authors' data and Platts. Map composed in ESRI ArcGIS.

- Greece receives its gas from diverse sources, and thus is resilient to the scenarios we analyse. It gets most of its gas from Russia (65.2%), from Turkey (16.4%) and LNG (18.4%);
- Ireland gets its gas from the UK and is unaffected by our scenarios, since the UK is a secure source in our model;
- Switzerland acts like a hub between South and Northern Europe, so it has very good access to network capacity;
- Ukraine is a major transit route for gas to Europe;
- Latvia and Finland have a relatively small population, good access to network capacity and are very close to Russia;
- Surprisingly, Belarus does better when Ukraine is removed from the network. The apparent contradiction is solved by realizing that Europe's supply from Russia has been historically built around Ukraine. Hence, Ukrainian routes have higher capacity and shorter routes to central Europe than routes that pass through Belarus. In contrast, when Ukraine is removed from the network, routes through Belarus become the first choice to supply central Europe;
- Belgium draws its high energy security from diversification of supply. It gets its gas from the Netherlands (42.5%), from Norway(48.7%, including LNG), Russia (2.8%), Germany (1.2%), and the UK (4.8%).

At urban level:

- Surprisingly, Rome seems to gain slightly from removing Libya. Rome is approximately in the middle of the Italy, and the country is supplied both from the South and from the North. When Libya is removed there is no need to transport gas from the South to the North of Italy and this frees capacity to bring more gas from the North to Rome;
- Unexpectedly, Berlin gains from the removal of Poland. Germany is transporting gas to Poland. When Poland is removed, the capacity that is freed can be used for German cities located close to the Polish border, including Berlin;
- Finally, Dublin is resilient to all scenarios because it gets all of its supply from the UK, and we do not have any scenario affecting the ability of UK to supply gas.

Table 1. Summary of notation

Indexes:	
i	Link index
j	Path index
m	Exporting country
n	Importing country
k	Node index in an exporting country
l	Node index in an importing country
Extracted at network level:	
η	Number of links in the network
d_i	Diameter of link i (units: in; vector with dimension η)
$c_i = 0.56 d_i^{2.5}$	Capacity of link i. The exponent 2.5 is found from data [20], and the prefactor is obtained by calibrating the present baseline scenario to match the T_{mn} flow matrix (units: Mm^3/year; vector with dimension η)
l_i	Length of link i (units: km; vector with dimension η)
B_{ij}	Link-path incidence matrix. $B_{ij} = 1$ if the link i belongs to the path r_j and $B_{ij} = 0$ otherwise (matrix with dimensions $\eta \times \rho$)
$b_i = \sum_{j=1}^{\rho} B_{ij}$	Number of paths that pass through link i (vector with dimension η)
Extracted at country level:	
ν	Number of countries
s_m	Number of (source) nodes in an exporting country m
t_n	Number of sink nodes in an importing country n
g_n	Number of Liquefied Natural Gas (LNG) terminals in an importing country n
T_{mn}	Volume of gas received by an importing country n from an exporting country m and LNG (matrix with dimensions: $(\eta + 1) \times \eta$)
$\widehat{T}_{mn} = \begin{cases} 1 & \text{if } T_{mn} \text{ is positive} \\ 0 & \text{otherwise} \end{cases}$	Binary matrix with zero entries if there is no transport between countries m and n
Z_{nl}	Population associated with sink node l of importing country n (matrix with dimensions $\nu\times$ [Number of Voronoi and urban sinks])
z_n	Population of an importing country n
Computed for the routing:	
ρ	Number of paths on the network
$r_{m,k,n,l}$	Path connecting source node k in an exporting country m with sink node l in an importing country n, where $k = 1, \ldots, \Phi_{mn}$ if m is an exporting country and $k = 1, \ldots, g_n$ if m is LNG
r_j	Paths are also indexed by an integer, to simplify the notation used in the congestion control algorithm (vector with dimension ρ)
$h_i = c_i/(1 + b_i)$	Share of capacity allocated to each path passing through link i at the beginning of the heuristic rerouting
D_{mnl}	Demand of sink l in an importing country n satisfied by an exporting country m
$\overline{D}_{mnl} = \left\lfloor \frac{D_{mnl}}{\min(D_{mnl})} \right\rfloor$	Number of identical paths between a source and sink pair, each having demand $\min(D_{mnl})$
$\widetilde{l_i} = \left(\frac{\langle h_i \rangle}{h_i}\right)^{\alpha} l_i$	Effective length of link i
$\overleftrightarrow{l_j}$	Effective length of path j (vector with dimension ρ)
Parameters:	
$\alpha = 0.03$	Exponent of $\langle h \rangle / h$ in equation (S4) (see also Figure S6)
$\Phi_{mn} = \begin{cases} \min(10, s_m) & \text{if } m \text{ is a country} \\ g_n & \text{if } m \text{ is LNG} \end{cases}$	If m is a country, we connect each sink node to the $\min(10, s_m)$ geographically closest nodes in an exporting country m (distance measured along network paths); if m is LNG, we connect each sink node to the g_n LNG terminals in an importing country n.
Congestion control algorithm:	
f_j	Path flow on path j (dimension ρ)
μ_i	Price on link i, and dual of f_j (vector with dimension η)
p_i	Price function on link i (vector with dimension η)
q_i	Inverse of p_i (vector with dimension η)
$L(f, \mu)$	Lagrangian function of the primal problem
$V(\mu)$	Lagrangian function of the dual problem

References

1. Bhaduri B, Bright E, Coleman P, Dobson J (2002) Landscan: Locating people is what matters. Geoinformatics 5: 34-37.

2. Angel S, Sheppard SC, Civco DL (2005) The dynamics of global urban expansion. Technical report, World Bank.

3. Schneider A, Friedl MA, Potere D (2009) A new map of global urban extent from modis satellite data. Environmental Research Letters 4: 044003.

4. International Energy Agency (2011) OECD - Natural Gas imports by origin. OECD/IEA.

5. Bettencourt LMA, Lobo J, Helbing D, Kuehnert C, West GB (2007) Growth, innovation, scaling, and the pace of life in cities. Proceedings of the National Academy of Sciences of the United States of America 104: 7301-7306.

6. Fehr E, Fischbacher U (2003) The nature of human altruism. Nature 425: 785-791.

7. Carvalho R, Buzna L, Just W, Helbing D, Arrowsmith DK (2012) Fair sharing of resources in a supply network with constraints. Physical Review E 85: 046101.

8. Schneider CM, Moreira AA, Andrade JS, Havlin S, Herrmann HJ (2011) Mitigation of malicious attacks on networks. Proceedings of the National Academy of Sciences of the United States of America 108: 3838-3841.

9. Bertsekas DP, Gallager R (1992) Data Networks. Prentice Hall, second edition.

10. Kelly FP, Maulloo AK, Tan DKH (1998) Rate control for communication networks: shadow prices, proportional fairness and stability. Journal of the Operational Research Society 49: 237-252.

11. Tan DKH (1999) Mathematical Models of Rate Control for Communication Networks. Ph.D. thesis.

12. Guler O (2010) Foundations of Optimization, volume 258. Berlin: Springer-Verlag Berlin.

13. Srikant R (2004) The Mathematics of Internet Congestion Control. Boston: Birkhäuser.

14. Kelly F (1997) Charging and rate control for elastic traffic. European Transactions on Telecommunications 8: 33-37.

15. Courant R, Hilbert D (1989) Methods of Mathematical Physics, volume 1. New York: Wiley-VCH.

16. Ball K (2008) Optimization and Lagrange Multipliers, in The Princeton Companion to Mathematics, New Jersey: Princeton University Press.

17. Boyd S, Vandenberghe L (2004) Convex Optimization. New York: Cambridge University Press.

18. Jacobson V (1988) Congestion avoidance and control. In: Proceedings of SIGCOMM 88. ACM, pp. 314-329.

19. Chiu DM, Jain R (1989) Analysis of the increase and decrease algorithms for congestion avoidance in computer-networks. Computer Networks and ISDN Systems 17: 1-14.

20. Carvalho R, Buzna L, Bono F, Gutierrez E, Just W, Arrowsmith DK (2009) Robustness of trans-European gas networks. Physical Review E 80: 016106.
